# Research and Remedies

**Published:** 1913-04-05

**Authors:** 


### RESEARCH AND REMEDIES.

### Diagnosis of Pregnancy by X-Rays.

Even the most expert and conscientious gynæcologists fail every now and then to diagnose pregnancy when they should do, or make the reverse mistake of diagnosing it in error. It may be doubted whether any specialist in this department with charge of a dozen or more hospital beds goes five years without being caught in one or other of these pitfalls. As a rule, large soft central fibroids are the commonest cause of the mistake, for they may mimic the consistency of the pregnant uterus so exactly that confusion of the two is quite easy. When such a doubtful enlargement has reached the size of a five or six months' pregnancy, the $x$-rays will give very valuable help in arriving at a correct diagnosis, though the appreciation of this fact has not yet dawned upon many gynæcologists, who will certainly come to realise it in a few years. According, however, to Edling, the $x$-rays can be made use of for the diagnosis of pregnancy at a much earlier stage than this. By careful attention to posture and by very short exposures to very intense secondary currents he professes to be able to diagnose pregnancy (or its absence) with the rays at the end of the second or beginning of the third month. The difficulties are the respiratory (maternal) and the active (fœtal) movements: thick uterine walls, as from fibroids, also may give rise to difficulty. It is most astonishing that the fœtal skeleton at this early stage of development should be capable of arresting the passage of the rays, but Edling assures us that this is the case. Other applications of this method of diagnosis which he has made include the detection of twin pregnancy (and its exclusion); and the diagnosis of malpresentations. Ectopic gestation, too, can, he believes, sometimes be diagnosed by this method. Injury to mother and fœtus is said to be effectively prevented by the use of Gehler's screen. The moral would seem to be that every lying-in hospital and gynæcological clinic ought to have an $x$-ray outfit and a competent radiologist to work it.

